# Progressive cognitive impairment after recovery from neuroinvasive and non-neuroinvasive *Listeria monocytogenes* infection

**DOI:** 10.3389/fimmu.2023.1146690

**Published:** 2023-04-18

**Authors:** Benjamin R. Cassidy, Sreemathi Logan, Julie A. Farley, Daniel B. Owen, William E. Sonntag, Douglas A. Drevets

**Affiliations:** ^1^ Department of Internal Medicine, College of Medicine, University of Oklahoma Health Sciences Center, Oklahoma, OK, United States; ^2^ Department of Biochemistry and Molecular Biology, College of Medicine, University of Oklahoma Health Sciences Center, Oklahoma, OK, United States

**Keywords:** *Listeria monocytogenes*, bacterial meningitis, CD8^+^ tissue-resident memory T lymphocytes, neuroinflammation, cognitive testing, cognitive dysfunction

## Abstract

**Background:**

Neuro-cognitive impairment is a deleterious complication of bacterial infections that is difficult to treat or prevent. *Listeria monocytogenes* (*Lm*) is a neuroinvasive bacterial pathogen and commonly used model organism for studying immune responses to infection. Antibiotic-treated mice that survive systemic *Lm* infection have increased numbers of CD8^+^ and CD4^+^ T-lymphocytes in the brain that include tissue resident memory (T_RM_) T cells, but post-infectious cognitive decline has not been demonstrated. We hypothesized that *Lm* infection would trigger cognitive decline in accord with increased numbers of recruited leukocytes.

**Methods:**

Male C57BL/6J mice (age 8 wks) were injected with neuroinvasive *Lm* 10403s, non-neuroinvasive Δ*hly* mutants, or sterile saline. All mice received antibiotics 2-16d post-injection (p.i.) and underwent cognitive testing 1 month (mo) or 4 mo p.i. using the Noldus PhenoTyper with Cognition Wall, a food reward-based discrimination procedure using automated home cage based observation and monitoring. After cognitive testing, brain leukocytes were quantified by flow cytometry.

**Results:**

Changes suggesting cognitive decline were observed 1 mo p.i. in both groups of infected mice compared with uninfected controls, but were more widespread and significantly worse 4 mo p.i. and most notably after *Lm* 10403s. Impairments were observed in learning, extinction of prior learning and distance moved. Infection with *Lm* 10403s, but not Δ*hly Lm*, significantly increased numbers of CD8^+^ and CD4^+^ T-lymphocytes, including populations expressing CD69 and T_RM_ cells, 1 mo p.i. Numbers of CD8^+^, CD69^+^CD8^+^ T-lymphocytes and CD8^+^ T_RM_ remained elevated at 4 mo p.i. but numbers of CD4^+^ cells returned to homeostatic levels. Higher numbers of brain CD8^+^ T-lymphocytes showed the strongest correlations with reduced cognitive performance.

**Conclusions:**

Systemic infection by neuroinvasive as well as non-neuroinvasive *Lm* triggers a progressive decline in cognitive impairment. Notably, the deficits are more profound after neuroinvasive infection that triggers long-term retention of CD8^+^ T-lymphocytes in the brain, than after non-neuroinvasive infection, which does not lead to retained cells in the brain. These results support the conclusion that systemic infections, particularly those that lead to brain leukocytosis trigger a progressive decline in cognitive function and implicate CD8^+^ T-lymphocytes, including CD8^+^T_RM_ in the etiology of this impairment.

## Introduction

Bacterial infections of the central nervous system (CNS) cause long-term neurological complications and cognitive dysfunction in children and adults (1–3). Despite a declining incidence of neurological diseases caused by meningitis in developed countries (4, 5), meningitis remains the 4^th^ leading cause of neurological disability-adjusted life-years globally (6). Additionally, recent data from a Finnish multicohort observational study found that individuals experiencing a CNS infection were 3-times more likely than uninfected individuals to develop dementia over a median follow-up period of 15.4 years (7). Host inflammatory responses are key drivers of post-infectious neurological dysfunction (8–11). Thus, suppressing brain inflammation with corticosteroids is the mainstay of currently approved adjunctive therapy (12, 13). Even though these drugs clearly benefit some patients, they have limited ability to improve neurocognitive outcomes (14–16). Continued research in models of bacterial brain infection is necessary to understand the etiology of pathological brain inflammation and ameliorate post-infectious cognitive dysfunction.

*Listeria monocytogenes* (*Lm*) is a food-borne, bacterial pathogen that invades the brain and frequently causes long-term neurological complications in survivors (17, 18). For example, a French national prospective study identified 818 cases of invasive listeriosis in which long-term neurological sequelae were present in 44% of patients that survived neurolisteriosis (19). This study also found significantly higher mortality in patients that were treated with adjunctive corticosteroids (OR 4.58 [1.50-13.98], p = 0.008). Smaller studies show sequelae are present in 16-30% of patients at hospital discharge (20, 21). *Lm* is also a tractable model organism and experimental *Lm* infection of mice is useful for studying CNS infections and host immune responses to bacterial infection (17, 18, 22). Nonetheless, if mice recovered from *Lm* infection exhibit long-term neurocognitive deficits, it would be a seminal finding as this model could be used to uncover pathological neuro-immune networks and identify targets for intervention.

To study brain inflammation after recovery from CNS *Lm* infection, we injected mice systemically with an otherwise lethal inoculum of *Lm*, then started antibiotic treatment 48 hrs later with the same drug used for human *Lm* infection (23). This model reflects the clinical situation of a patient receiving treatment after developing neuroinvasive *Lm*. Analysis of microglial gene expression and brain leukocyte influxes 3d post-injection (p.i.) showed marked upregulation of IFN-related genes in microglia and influxes of Ly6C^hi^ monocytes, followed at 7d p.i. by large numbers of CD8^+^ T-lymphocytes (23). Importantly, numbers of CD8^+^ T-lymphocytes cells in the brain expressing a phenotype consistent with tissue-resident memory (T_RM_) cells remained significantly elevated 1 mo p.i. and produced IFN-γ and TNF upon stimulation (23, 24). These findings are notable as anti-viral CD8^+^ T-lymphocytes recruited to the brain during acute viral encephalitis can provoke neuronal damage in an IFN-γ-dependent mechanism *via* activated microglia that eliminate synapses (25, 26). Additionally, CD8^+^ T_RM_ have emerged as critical cells that instigate neurological injury in humans and in mouse models of neuroinflammation, including when instigated by infection (27, 28). These cells are recruited into the CNS during infection with bacteria, protozoans, and viruses and provide immune surveillance and rapid pathogen removal upon re-infection (24, 29–33). Tissue retention in extravascular niches is accomplished in part by surface antigens including CD49a, CD69, and CD103, pathological exert actions are due to bystander activation of parenchymal cells *via* IFN-γ signaling and through antigen-specific activation, e.g. during re-infection (28).

The goal of this study was to test the hypothesis that *Lm* infection alters cognitive function in mice in a manner commensurate with the degree of brain inflammation. To distinguish specific effects of CNS infection/inflammation on cognition from those caused by peripheral infection, we compared results using the neuroinvasive *Lm* strain 10403s with a gene-deletion mutant that lacks the *hly* gene (Δ*hly*) encoding listeriolysin O (34). This mutant does not escape from phagosomes, replicate intracellularly, or activate cytosolic response mechanisms (35, 36). Moreover, systemic infection with Δ*hly Lm* mutants does not cause brain infection, widespread upregulation of brain inflammatory genes, or CNS influxes of blood leukocytes (23, 37). Cognitive function was tested using the Ethovision PhenoTyper with Cognition Wall, a food reward-based discrimination procedure in an Automated Home Cage based observation and Data Analysis (AHCoDA) system with continuous monitoring (38, 39).

Results presented here show mice infected with virulent *Lm*, as well as with Δ*hly Lm*, had significantly worse cognitive outcomes 4 mo after infection/treatment. These changes included decreased learning, impaired ability to extinguish prior learning, and a decline in overall movement during the observation period. Concomitant analysis of brain leukocytes showed that increased numbers of CD4^+^ and CD8^+^ T-lymphocytes were present in the brain 1 mo p.i., but only CD8^+^ T-lymphocytes remained increased 4 mo p.i. when cognitive changes were most pronounced. These data are in accord with the hypothesis that CD8^+^ T-lymphocytes recruited to the brain during infection trigger cognitive decline.

## Materials and methods

### Antibodies

Fluorochrome-conjugated mAb (clone, fluorochrome) and isotype-matched control mAb were purchased from BD Pharmingen (San Diego, CA): CD62L (MEL-14, BV510), CD44 (IM7, PE-CF594), Biolegend (San Diego, CA): CD11b (M1/70, BV421), CD3 (17A2, PE), CD8a (53-6.7, Alexa Fluor 488), CD4 (RM4-5/GK1.5, BV605 and BV785), CD45 (30-F11, PE/Cy7), CX_3_CR1 (SA011F11, BV605), Ly-6G (1A8, BV510), Ly-6C (HK1.4, PerCP/Cy5.5), CD69 (H1.2F3, BV711), and CD103 (2E7, APC).

### Bacteria

*Lm* strain 10403s was purchased from the American Type Culture Collection (Manassas, VA). The Δ*hly Lm* mutant DP-L2161, which is constructed from the 10403s parent strain, was a kind gift from D. Portnoy (Univ. of California, Berkeley, CA) (34). Bacteria were stored in brain-heart infusion (BHI) broth (Difco, Detroit, MI) at 10^9^ CFU/ml at -80°C. For experiments, the stock culture was diluted 1:10,000 into BHI and incubated with shaking overnight at 37°C.

### Animal infection and antibiotic treatment

This study was approved by the Institutional Animal Care and Use Committee (IACUC) of the University of Oklahoma HSC. Male C57BL/6J mice purchased from Jackson Laboratories (Bar Harbor, ME) were 8 wks old at time of infection or injection. Mice from multiple litters of similar age were randomly assigned to be infected i.p. with 500 µL PBS alone or PBS containing the indicated dose/strain of *Lm*. Each group of infected mice was injected on the same day to reduce variability and received amoxicillin (2 mg/mL final concentration) in drinking water *ad libitum* as previously described (23, 24). Mice were randomly assigned to undergo cognitive and immunological analysis at 1 mo or 4 mo p.i. There were no mortalities. This study used only male mice as it was not powered to study potential confounding effects of estrous cycles on behavioral analyses (40).

### Cognitive testing

**Figure 1:**
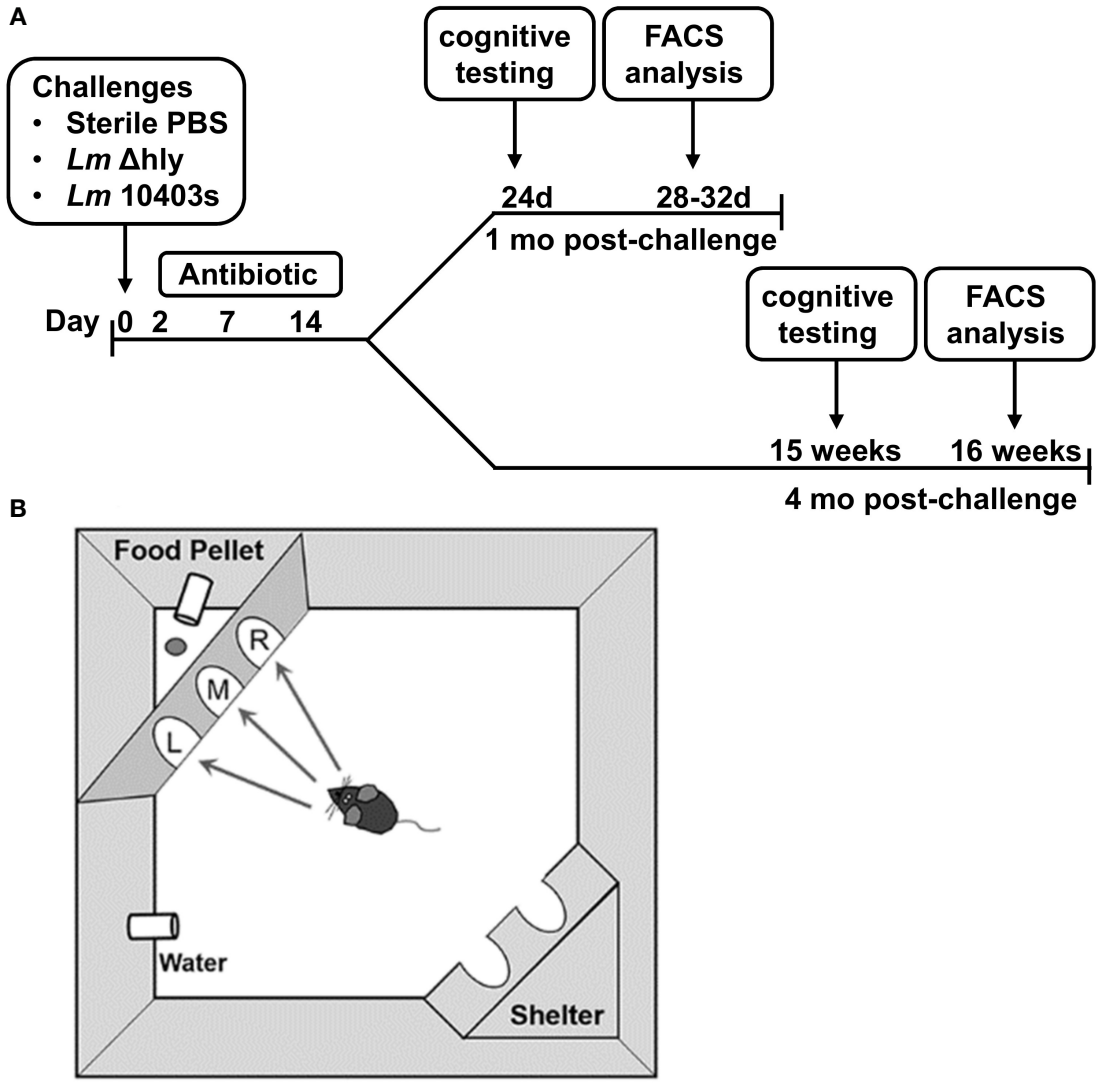
Timeline of *Lm* infection and graphical description of cognitive testing. C57BL/6J mice (age 2 mo) were injected i.p. with avirulent *Lm* Δ*hly* (3.0 x 10^7^ CFU, 1 mo n=9, 4 mo n=8), neuroinvasive *Lm* 10403s (3.2 x 10^6^ CFU, 1 mo n=11, 4 mo n=5), or with sterile PBS as uninfected controls (1 mo n=12, 4 mo n=7). All mice received antibiotics beginning 2d after injection with the indicated bacteria or sterile PBS. Mice were split into 2 cohorts with half undergoing early cognitive testing and the other half undergoing late cognitive testing. **(A)** Cognitive testing was performed using the Ethovision PhenoTyper with Cognition Wall **(B)**. During hours 1-48 (initial Discrimination period), the mouse was rewarded on a fixed ratio schedule of 1 food pellet per 5 entries through the left most entry of the cognition wall. The algorithm is reversed at hour 49 and from hours 49-89 (reversal phase), the mouse learned to enter the right most entry to receive a reward in the same reward schedule. Movement was tracked using an IR camera located above the cage using Noldus Ethovision software.

To determine if cognitive function was impaired after *Lm* infection, we infected 2 mo old male C57BL/6J mice i.p. with neuroinvasive *Lm* strain 10403s (3.2 x 10^6^ CFU/mouse), a lethal dose without antibiotic treatment. For a comparison, other mice were infected i.p. with 3.0 x 10^7^ CFU/mouse of a gene-deletion mutant derived from 10403s lacking *hly* (Δ*hly*), which encodes for listeriolysin O (34). This mutant does not escape from phagosomes into the cytosol or replicate intracellularly, not does it invade the brain or promote cellular influxes into it (23, 35–37). A third group of mice were injected i.p. with PBS and remained uninfected. All mice received the same antibiotic regimen regardless of infection status. One cohort of each treatment group (*Lm* 10403s-infected, Δ*hly Lm*-infected, uninfected) underwent cognitive testing in the Ethovision PhenoTyper with Cognition Wall 24d p.i. (referred to as 1 mo p.i.) whereas the remainder were tested 15 wks p.i. (referred to as 4 mo p.i.). A flow diagram is displayed in **Figure 1A**.

Cognitive testing was performed using the Ethovision PhenoTyper with Cognition Wall (Model 3000, Noldus Information Technology, The Netherlands). The PhenoTyper with Cognition Wall is an automated home-cage device that measures movement and cognitive ability non-invasively and without animal handling (**Figure 1B**) (38, 39). Four days prior to testing, the mice were individually housed and given access to food pellets (Dustless Precision Rodent Pellets, F05684, Bio-Serv, Flemington, NJ) used in the experiment for adaptation to the food. Mice were monitored continuously over four (4) 12 hr light: dark periods during which diurnal spontaneous movement is continuously tracked. The mouse learned to make the left most entry to receive a food reward pellet in the initial discrimination (acquisition) period (hours 1-48). Food was dispensed using a Fixed Ratio (FR) 5 schedule in which the mouse was required to make 5 left entries to receive 1 food pellet. During the Reversal period (hours 49-89), the algorithm was changed such that the mouse has to enter through the right most entry to receive a food reward, also using the FR5 schedule. Thus, the mouse must extinguish the previous memory to receive a food reward using the same reward schedule. Ethovision software version 16 (Noldus, Netherlands) was used to record the activity of the mice, which was tracked *via* an IR camera located above the cage. Pre- and post-behavioral testing weights were recorded, and the data exported as delimited text files (1 per mouse) and processed using R programming to represent values per hour. Extractable data include movement, feeding, independent learning index, cumulative learning index, entry choice, and entries to criterion data table. Calculations for cumulative learning index and time/entries to criterion are described in the section below entitled *Statistical Analysis and Calculations*.

Due to instrument error, behavioral data was not recorded for 4 10403s-infected mice, 2 Δ*hly*-infected mice, and 2 uninfected mice. Additionally, one Δ*hly*-infected mouse in the 4 mo group was excluded from data analysis due to failure to engage in the initial learning task. All mice were euthanized within one week of completing cognitive testing for immunological analysis.

### Euthanasia, necropsy and brain digestion

After cognitive testing, analysis of brain leukocytes was performed as previously described (24). Briefly, mice were euthanized by CO_2_ asphyxiation and exsanguinated *via* femoral vein cut-down, then were perfused trans-cardially with 25 mL iced, sterile PBS containing 2 U/ml heparin. Brains were removed aseptically after perfusion and digested enzymatically for 45 min at 37°C in Miltenyi C tubes (Miltenyi Biotec, San Diego, CA) containing 0.5 mg/mL collagenase IV and 0.025 mg/mL DNAse I in RPMI-1640 (ATCC, Manassas, VA) plus 1% penicillin/streptomycin and 10% fetal bovine serum (FBS). The disaggregated organ was passed through a 70 µm cell strainer, which was rinsed with 10 mL HBSS without Ca^++^/Mg^++^ (Lonza, Basel, Switzerland), then centrifuged at 300x g for 10 min at room temperature. The supernatant was discarded and the cells were suspended in 30% Percoll (GE Healthcare, Chicago, IL) in a 15 mL conical tube then centrifuged at 700x g at RT for 10 min to remove myelin. The cell pellet was washed once with PBS + 0.5% BSA and erythrocytes were lysed with RBC Lysis Buffer (Life Technologies Corp., Carlsbad, CA) for 5 min at RT. The cells were washed twice with PBS + 0.5% BSA at 300x g for 10 min at 4°C then re-suspended in 3 mL of PBS + 0.5% BSA and counted (Countess II FL Automated Cell Counter, Life Technologies Corp.).

### Flow cytometry

Cells were incubated for 20 min on ice with 2 uL of anti-CD16/32 TruStain fcX (BioLegend, San Diego, CA) plus 10 uL of Brilliant Stain Buffer Plus (BD Biosciences, Franklin Lakes, NJ). Next, fluorochrome-labeled mAb were added, and the cells were incubated in the dark at RT for 20 min then were washed twice with 3 mL FACS buffer (1x PBS + 0.5% BSA + 0.1% NaN_3_). Cells were then fixed with 200 uL IC Fixation buffer (Life Technologies Corp.) at RT for 20 min in the dark, then washed again with 3 mL FACS buffer, and stored at 4°C in the dark until analyzed. Flow cytometry was performed on a Stratedigm S1200Ex (Stratedigm Inc, San Jose, CA) and results were analyzed with CellCapTure software (Stratedigm).

### Statistical analysis and calculations

The Prism 9 statistical suite (GraphPad Software, San Diego, CA) was used for aimple statistical analysis and graphing. Flow cytometry data were analyzed by 1-way ANOVA with Tukey’s post-test or by non-parametric Kruskal-Wallis test with Dunn’s post-test among groups compared at the same time point. A two-tailed Student’s *t* test assuming equal variance was used when 2 groups that received the same treatment were compared across two time points. Analyses of cognitive performance that varied by infection status and testing period were performed by 2-way ANOVA corrected for multiple comparisons by controlling the False Discovery Rate for discoveries at Q < 0.05 with the Benjamini, Krieger and Yekutieli method (41). Achievement of time to 80% criterion and entries to 80% criterion were defined as the time or entry at which an individual mouse achieved 24 correct entries out of the past 30 entries. Significance was calculated *via* pairwise application of the log-rank test. With all tests a p < 0.05 was considered significant. Cumulative Learning Index is calculated as ( $\frac{Correct-Incorrect}{Correct+Incorrect}$ ) with *Correct* indicating total number of correct entries and *Incorrect* indicating total number of incorrect entries. The index was calculated at each hour and reset at hour 49 with the start of the reversal phase. Cognitive flexibility was calculated as the gain in cumulative learning index between hours 51 and 61 (during the first dark cycle of the reversal phase). Maximum learning was defined as the cumulative learning index attained at hour 89, corresponding to the end of cognitive testing. Statistical significance was evaluated using the independent learning index values using JMP (v15.2.0, SAS Inc, Cary, NC) and cumulative values were calculated.

## Results

### *Lm* infection impairs learning

**Figure 2:**
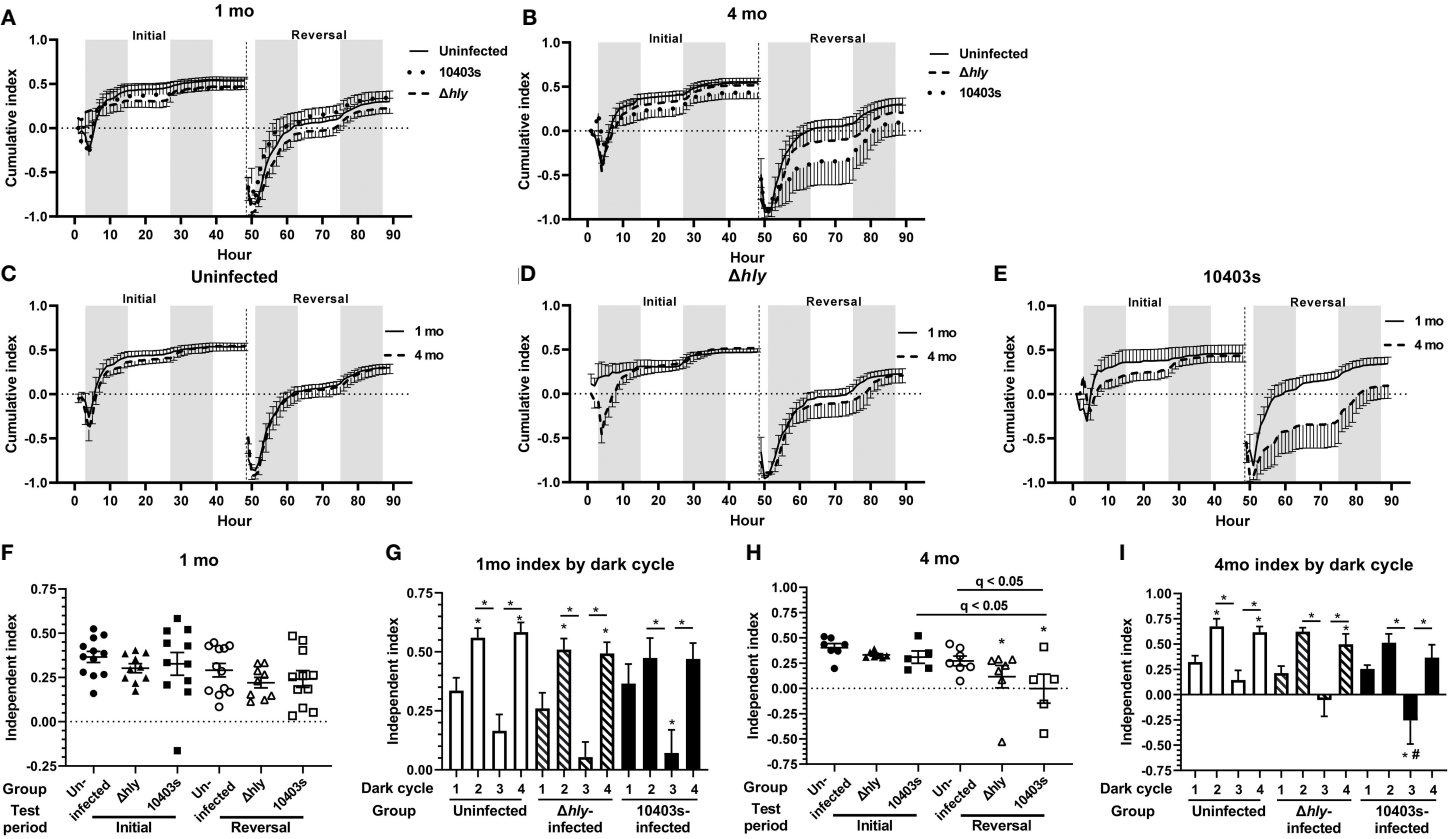
*Lm* 10403s-infected mice display impaired cognitive performance 4 mo after infection. Panels A, B, display cumulative learning index for Initial and Reversal phases calculated at each time point, with the index resetting at the beginning of the Reversal phase (hr 49). Dark (shading) and light (no shading) cycles are indicated. Panels A and B show cumulative indices (+/- SEM) for all Uninfected, Δ*hly*-infected and 10403s-infected at 1 mo **(A)** and 4 mo **(B)** p.i. Panels C-E show cumulative indices at 1 mo (solid line) and 4 mo (broken line) post p.i. for individual treatment groups, Uninfected **(C)**, *Lm* Δ*hly*-infected **(D)**, and *Lm* 10403s-infected **(E)**. Independent learning indices at 1 mo **(F, G)** and 4 mo p.i. **(H, I)** show individual mice **(F, H)** represented as uninfected (●○), *Lm* Δ*hly*-infected (▲Δ), and *Lm* 10403s-infected (■□). Lines show the group mean +/- SEM. Panels G & H show the mean (SEM) Independent Learning index according to dark cycles 1-4 in the initial (cycles 1 & 2) and reversal (cycles 3 & 4) periods. Discoveries (q < 0.05) by 1-way ANOVA in panels F and H comparing all groups with uninfected mice in the initial phase are indicated by (*), discoveries and q values between other groups are shown. Discoveries (q < 0.05) in panels G and I comparing the same dark cycle between *Lm* Δ*hly*-infected and 10403s-infected mice with uninfected mice are indicated by (#).

Cognitive function was evaluated initially by determining a cumulative learning index (**Figures 2A–E**). This measurement allows a visual assessment of the rate of learning (i.e., the slope of the curve during initial discrimination and reversal learning) as well as the maximum learning capacity during the specific testing period as previously described (38, 39). Differences among the groups were relatively minor in both initial and reversal phases at 1 mo p.i. (**Figure 2A**). However, by 4 mo p.i., 10403s-infected mice clearly diverged from the other groups (**Figure 2B**). Differences in performance are more evident when results at 1 mo and 4 mo p.i. of each treatment group are directly compared. This shows cumulative learning indices at 1 and 4 mo of uninfected mice overlap each over, whereas minor variances are observed for Δ*hly Lm*-infected mice (**Figures 2C, D**). In contrast, 1 and 4 mo indices from mice recovered from *Lm* 10403s infection diverged, particularly during the reversal phase (**Figure 2E**). Infection with *Lm*, irrespective of virulence, impaired cognitive performance in the reversal phase compared to uninfected controls (**Figures 2F, H**, and **Supplemental Figure 2**). Additionally, at 4 mo p.i. 10403s-infected mice performed worse in the reversal phase than they did in the initial phase, and when compared with uninfected mice in the reversal phase (**Figure 2H**). These results suggest that *Lm* infected-mice display impaired learning compared with antibiotic-treated control animals. Furthermore, the degree of impairment was more noticeable at 4 mo p.i. compared with 1 mo p.i. and in *Lm* 10403s-infected mice than in Δ*hly Lm*-infected mice.

As previously shown in this system (38, 39), mice are most active during dark cycles and the same was found in all groups in this study (**Supplemental Figure 1**). Thus, for a more detailed view of cognitive decline caused by infection, we analyzed the independent learning index in each of the dark cycles incorporated into the initial (cycles 1 &2) and reversal (cycles 3 & 4) phases at 1 mo p.i. (**Figure 2G**) and 4 mo p.i. (**Figure 2I**). These analysis showed that each group of mice had its lowest performance in dark cycle 3, the beginning of the reversal phase. Notably, *Lm* 10403s-infected mice, but not Δ*hly Lm*-infected mice, performed statistically worse in dark cycle 3 than did uninfected mice in the same cycle (**Figure 2I**). Additionally, results at 4 mo each show that uninfected and Δ*hly Lm*-infected mice significantly increased their learning indices in dark cycle 4 compared with dark cycle 1, whereas there was no such increase for 10403s-infected mice (**Figure 2I**). These data support the notion infection with *Lm* 10403s inhibits learning to a greater degree than infection with Δ*hly Lm*.

Given the relatively small “n” of 10403s-infected mice at 4 mo p.i., we were interested in determining the degree to which these results would be reproducible. For this, we compared results at 1 mo p.i. of the mice reported above (n=11), which were infected with 3.2 x 10^6^ CFU *Lm* 10403s, with a separate cohort of C57BL/6J mice of the same age (n=10) infected i.p. with 6.2 x 10^5^ CFU of *Lm* 10403s and also analyzed at 1 mo p.i. (**Supplemental Figure 2**). Results show highly comparable independent indices between the different cohorts (**Supplemental Figure 2A**). There were no statistical differences between the cohorts in independent indices from initial or reversal phases although cohort 1 had a greater variance in the initial test period than did cohort 2. Data in **Supplemental Figure 2B** show that combining the two cohorts of *Lm* 10403s-infected mice gives additional statistical power to detect a significant difference in the independent indices of infected mice measured 1 mo post-infection compared with uninfected controls. When analyzed by individual dark cycles, there were no differences in cohorts between the same cycle, e.g. cohort 1 cycle 1 versus cohort 2 cycle 1 (**Supplemental Figure 2C**). Combining individual dark cycle results of cohorts 1 and 2 did not identify new findings when this groups was compared to other treatment groups (**Supplemental Figure 2D**). These data show there is a high degree of reproducibility between results of different cohorts and increase confidence in the data derived from *Lm-*infected mice at 4 mo p.i. despite their small numbers. Subsequent analyses at 1 month use exclusively cohort 1 mice because these animals received the same inoculum of *Lm* 10403s as did those studied at 4 mo p.i.

**Figure 3:**
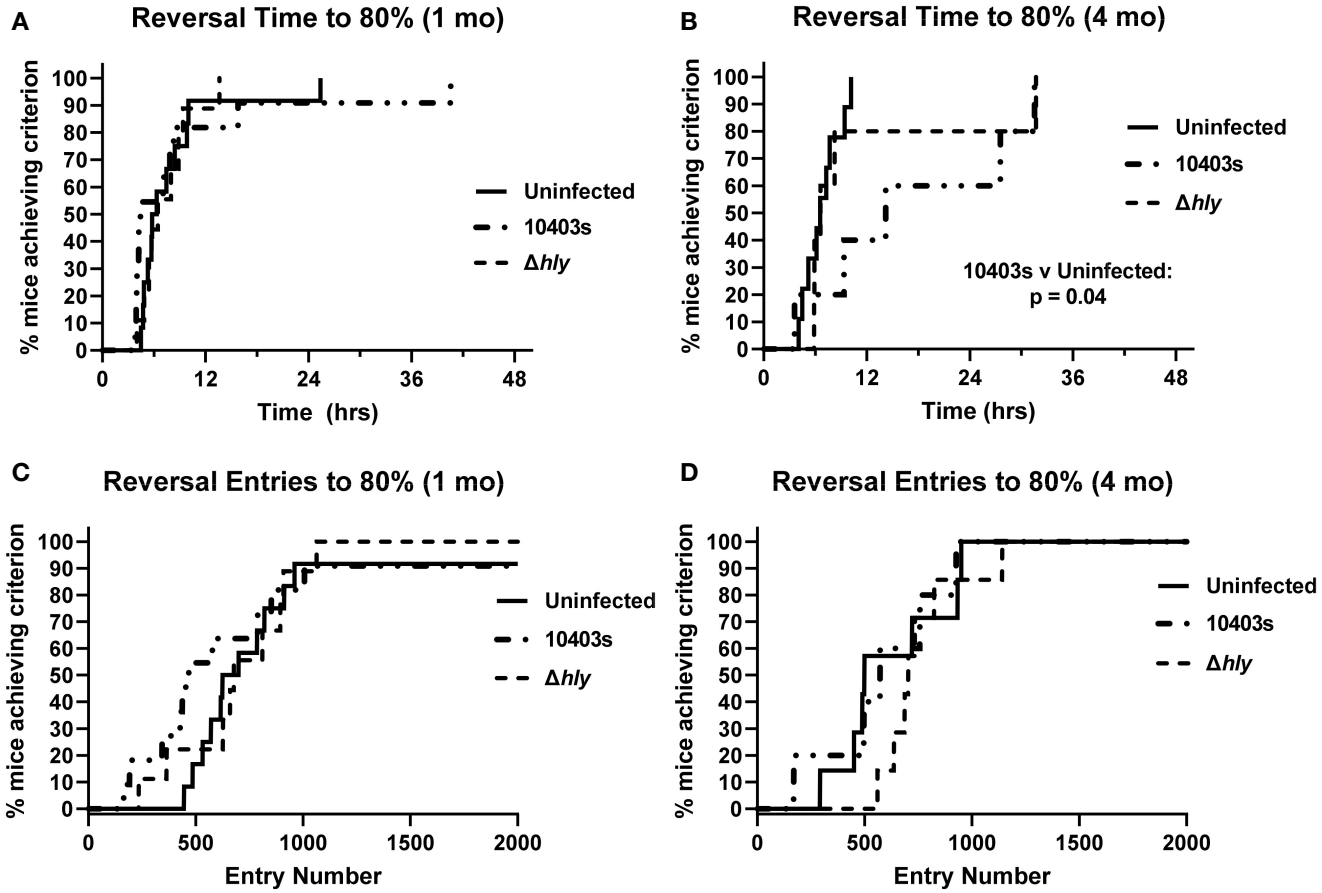
Infection with virulent *Lm* prolongs time to achieve criterion during reversal phase. Percent correct entries during the trailing 30 trials were calculated and the time or entries necessary to reach an 80% success rate calculated. Panels show the time (hrs) required **(A, B)**, and total number of entries **(C, D)** required versus the % of Uninfected (thin line), Δ*hly Lm*-infected (dashed line), and 10403s-infected (dotted line) mice. All panels display achievement of criteria within the reversal phase. Significance of Kaplan-Meier plots were calculated using a pairwise application of the log-rank test and significant differences are presented.

Next, we measured the number of correct entries the animal makes through the cognition wall and the time required to achieve a pre-established criterion of success, specifically the percentage correct entries. The selected criteria for entries required that the animal successfully learn each task based on a success rate of 80% over the trailing 30 entries as previously described (38, 42). Results presented in **Figure 3** show that 10403s-infected mice required significantly more time to achieve the 80% criterion at 4 mo p.i. than did uninfected mice in the reversal phase (**Figure 3B**) whereas there was no difference at 1 mo p.i. (**Figure 3A**). There were no differences in the number of entries performed (**Figures 3C, D**) despite 10403s-infected mice requiring more time to achieve criterion at 4 mo (**Figure 3D**). In contrast, there were no differences among any group in the initial phase for time or number of entries required to reach the 80% criterion whether measured at 1 or 4 mo p.i. (data not shown).

### *Lm* infection impairs extinction of prior learning at 4 mo but not at 1 mo p.i.

**Figure 4:**
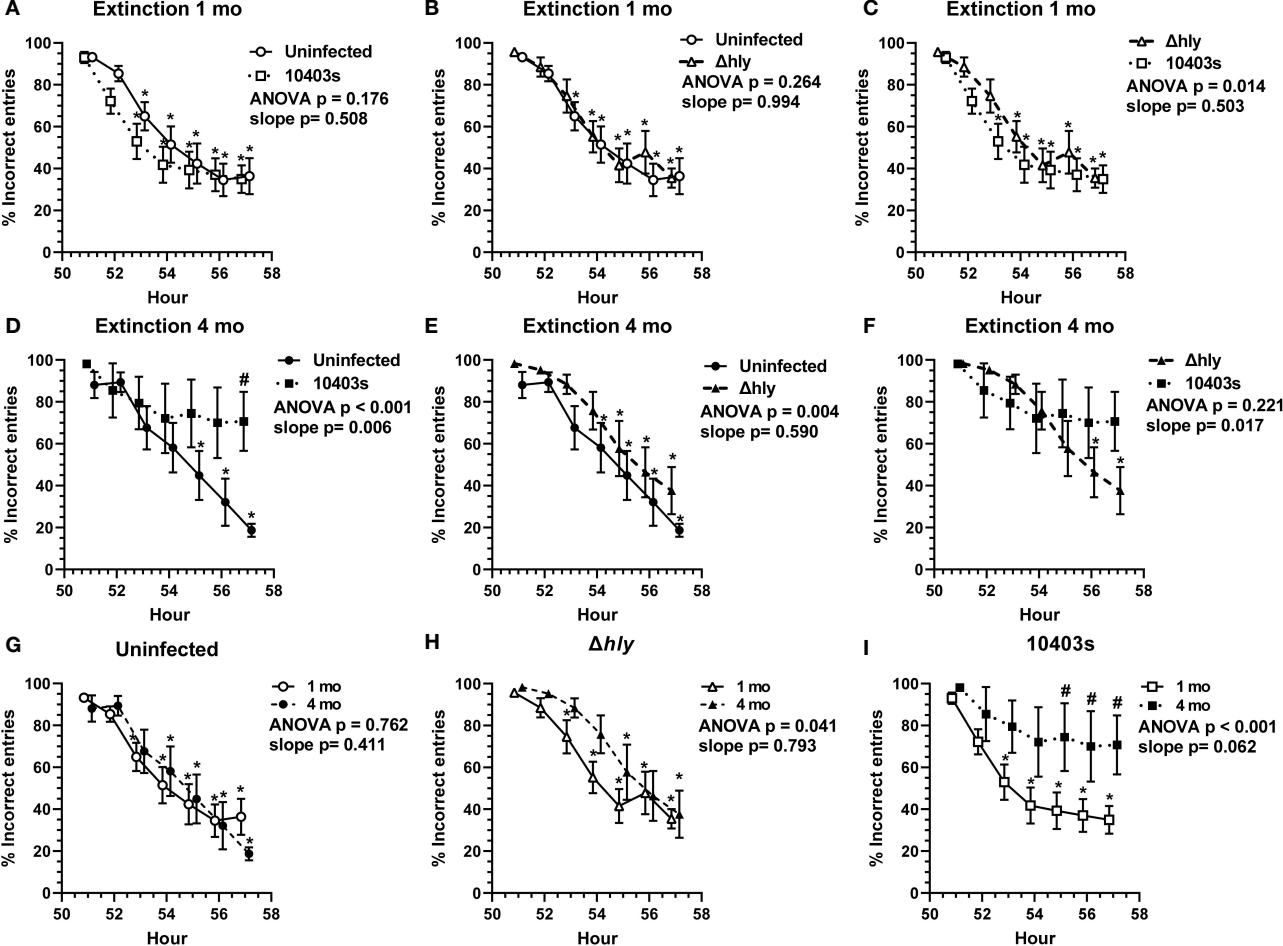
*Lm* infection impairs ability to extinguish prior learning. The % incorrect entries (#left door + #middle door)/total # entries x 100 of mice from hours 51-57 at the beginning of the reversal phase were analyzed to measure the animal’s ability to extinguish prior learning. Figures show mean +/- SEM % incorrect entries measured 1 mo **(A–C)** and 4 mo **(D–F)** after infection or injection, or according to treatment groups at 1 mo and 4 mo **(G–I)** from uninfected mice (● n=11, ○ n=7), and from mice recovered from infection with *Lm* Δ*hly* (▲ n=9, Δ n=7) or *Lm* 10403s (■ n=11, □ n=5). Symbols at the same time are offset for clarity. p values from 2-way ANOVA comparing extinction curves are given. 2-way ANOVA was used to identify discoveries (q < 0.05) between comparator groups at the same time point are indicated by (#). Discoveries (q < 0.05) in the same test group compared to its own hr 51 are shown by (*). Significant differences between slopes of regressions lines were determined by Analysis of Covariance (ANCOVA) to calculate a two-tailed P value.

Another key measurement of cognitive function is the ability to extinguish prior learning. We measured this by quantifying the % incorrect (% left door + % middle door) entries from hours 51-57 in dark cycle 3, immediately after the food reward algorithm changed from receiving a reward through the left door to right door. Extinction curves at 1 mo from uninfected mice and mice recovered from 10403s and Δ*hly Lm* infection were not different (**Figures 4A, B**). However, comparison of *Lm* 10403s-infected and Δ*hly Lm*-infected mice did show that 10403s-infected mice performed better, i.e. could extinguish prior learning more rapidly, than did mice infected with Δ*hly Lm* mutants (**Figure 4C**). Nonetheless, the curves were highly similar. Additionally, each test group showed significant reduction in % incorrect entries by the 53 or 54hr time points (**Figures 4A–C**). When analyzed 4 mo after infection, *Lm* 10403s infected mice performed significantly worse than did uninfected mice (**Figure 4D**). *Lm* 10403s infected mice did not achieve a significantly lower % correct entries during the test period whereas uninfected mice did (**Figure 4D**). In contrast, although Δ*hly Lm*-infected mice also performed worse than did uninfected mice, they did significantly reduce the % incorrect entries during the test period and the shape of the extinction curve was similar to that of uninfected mice (**Figure 4E**). Comparison of *Lm* 10403s infected and Δ*hly Lm*-infected mice showed no significant difference by ANOVA but did have significantly different slopes suggesting more rapid extinction in Δ*hly Lm*-infected mice than in 10403s-infected mice (**Figure 4F**).

Next, we compared the performances of each group at 1 mo with the same groups performance at 4 mo p.i. (**Figures 4G–I**). There were no differences between the performances of uninfected mice (**Figure 4G**). Δ*hly*-infected mice performed worse at 4 mo although they did achieve the same % of incorrect entries during the test period as at 1 mo (**Figure 4H**). In contrast, the performance of *Lm* 10403s-infected mice was substantially worse at 4 mo compared with 1 mo (**Figure 4I**). As noted previously, *Lm* 10403s-infected mice did not significantly lower their % incorrect entries during the observation period at 4 mo after infection despite having done so 1 mo after infection (**Figure 4I**). Moreover, the % incorrect entries at individual time points of 55, 56, and 57 hrs were worse (q <0.05) at 4 mo than at 1 mo post-infection. These data indicate that *Lm* infection leads to impairments in extinction. Similar to results in the learning index (**Figures 2F–H**), cognitive declines observed in Δ*hly Lm*-infected mice were less pronounced than in 10403s-infected mice.

Cognitive flexibility is the ability to extinguish one learned memory while learning a new task (43). Here, it was measured as the increase in the cumulative learning index during the hours 51-61 of the first dark cycle of the reversal phase. Measurements of cognitive flexibility and extinction differ in that cognitive flexibility takes acquisition of the new task into account whereas extinction does not. Data in **Supplemental Figures 3A, B** show no statistically significant differences among the groups when only a cohort 1 (n=11) of *Lm*-10403s-infected mice at 1 mo p.i. were included in the analysis. However, when this number was increased to include both cohorts 1 and 2 (n=21) mice recovered from *Lm* 10403s infection showed decreased cognitive flexibility at 4 mo p.i. compared with 1 mo p.i. (p = 0.047) (**Supplemental Figure 3C**). A similar pattern was observed in maximum learning attained by the end of the reversal phase, with 10403s-infected mice displaying a trend (p = 0.077) toward decreased maximum learning at 4 mo p.i. (**Supplemental Figure 3D**).

### Distance moved in a home-cage setting declines after *Lm* infection

**Figure 5:**
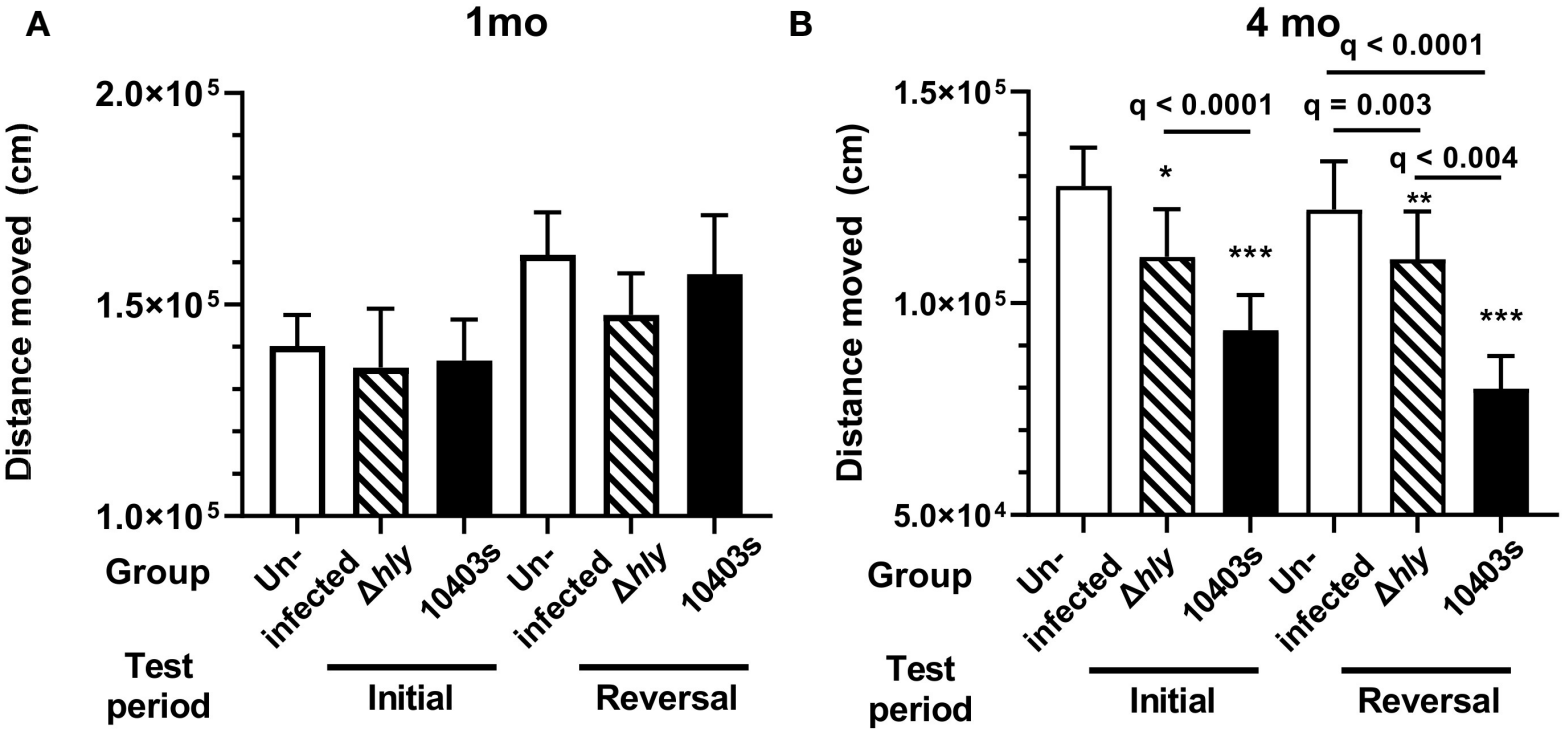
*Lm* infection reduces spontaneous distance moved. Movement over 89h was measured in uninfected mice (open bars), mice infected with *Lm* Δ*hly* mutants (hatched bars) and *Lm* 10403s (solid bars) during initial and reversal phases at 1 mo p.i. **(A)** and 4 mo p.i. **(B)**. Columns in panels represent mean + SEM distance moved in initial and reversal phases. 2-way ANOVA was used to identify discoveries (q < 0.05) between Δ*hly Lm* and *Lm* 10403s compared with uninfected mice in the same period are shown as *q < 0.05, **q < 0.01, ***q < 0.001. Discovery q values between other groups are given.

Human studies show that distance moved, as measured by daily steps, correlates positively with several key health indicators including lower all-cause mortality, lower Aβ burden and Aβ-related cognitive decline, and lower risk of all-cause dementia (44–47). Thus, we were interested in the effect of *Lm* infection on distance moved by mice during the testing periods. When tested 1 mo after infection there were no differences between the groups in the distance moved in the initial or reversal phases (**Figure 5A**). In contrast, when measured at 4 mo p.i., *Lm*-infected mice moved statistically less than did uninfected mice in both initial and reversal periods (**Figure 5B**). Moreover, 10403s-infected mice moved less than did Δ*hly*-infected mice in both periods. It is possible that decreased movement, rather than decreased cognitive function, could explain results in **Figure 3B** in which *Lm* 10403s-infected mice took longer to achieve the test criterion. To test this, we correlated distance moved in the reversal period with time to the 80% criterion and number of entries to reach the 80% criterion in the same period and found correlation p values of >0.39 and >0.45, respectively, using both Pearson and Spearman methods. Collectively, results showing *Lm*-infected mice have decreased movement, particularly evident 4 mo p.i. and to a more severe degree in *Lm* 10403s-infected mice than in Δ*hly*-infected, mice are in accord with the overall finding that *Lm* infection triggers cognitive decline.

### Dynamic changes in brain leukocyte populations after *Lm* infection

**Figure 6:**
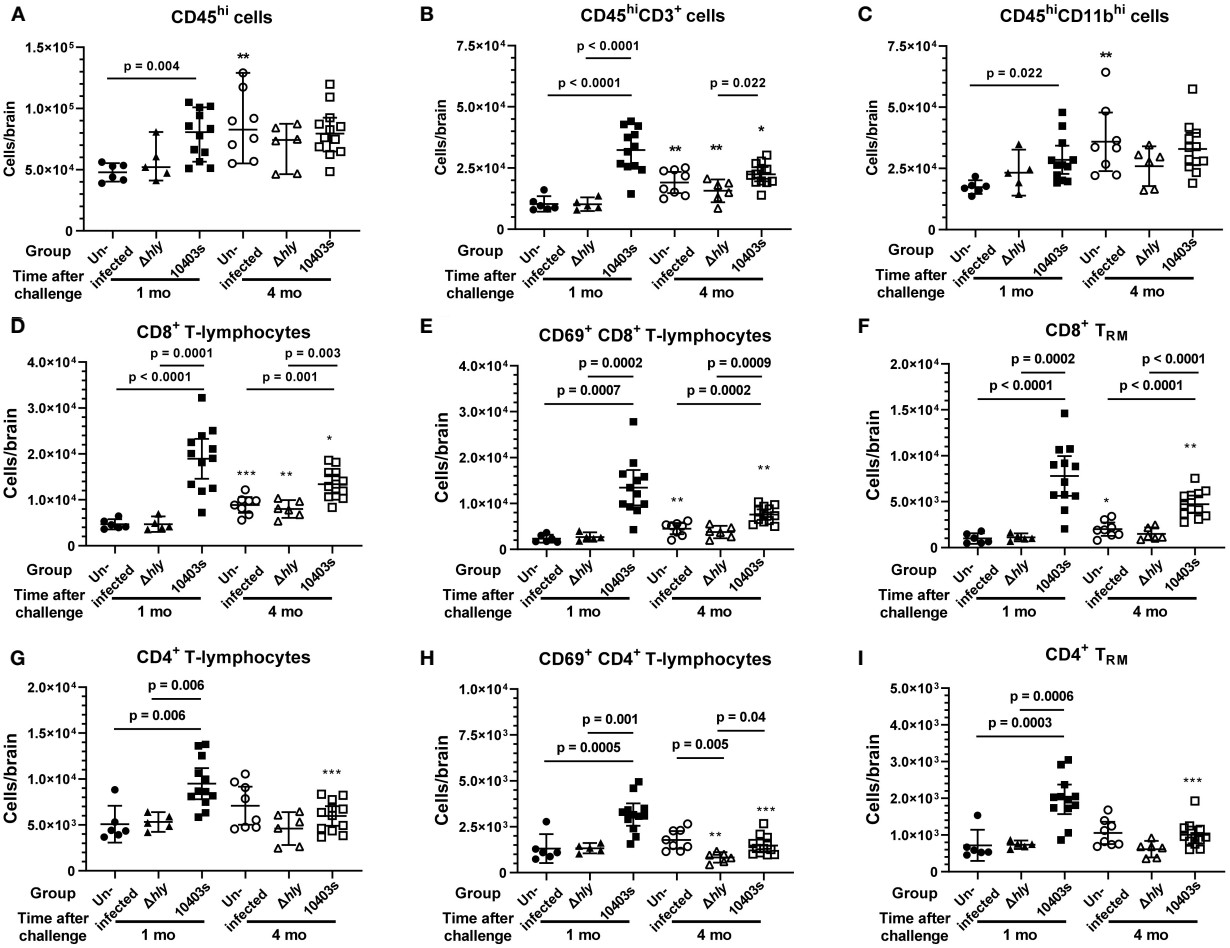
Analysis of bone marrow-derived leukocytes in brains of mice 1 and 4 mo after injection with neurovirulent *Lm* or avirulent Δ*hly* mutants. Brain leukocytes were collected from uninfected mice (●, ○) and from mice infected with *Lm* Δ*hly* mutants (▲, Δ) and *Lm* 10403s (■, □) described in **Figure 1** at 1 and 4 mo after infection, along with additional mice from the same treatment cohorts for which cognitive data were not collected. Leukocytes were collected from perfused brains and analyzed by flow cytometry and are shown as total CD45hi cells **(A)**, total lymphocytes **(B)**, total myeloid cells **(C)**, CD8+ lineage lymphocytes **(D–F)**, and CD4+ positive lineage lymphocytes **(G–I)**. Symbols indicate individual mice and lines represent group mean +/- 95% CI. Numbers of samples analyzed 1 mo and 4 mo after infection, respectively, are uninfected (6, 8), *Lm* Δ*hly* infected (5, 6), and *Lm* 10403s infected (12, 12). Significance within time points were calculated *via* 1-way ANOVA with Tukey’s post-test are given. Significance between the same challenge groups at 1 and 4 mo were calculated *via* 2-tailed Student’s *t*-test. *p < 0.05, **p < 0.01, ***p < 0.001.

In this model of *Lm* infection, large numbers of IFN-activated T-lymphocytes, as well as smaller numbers of neutrophils and Ly6C^hi^ monocytes, are recruited to the brain within 7d p.i., and numbers of CD4^+^ and CD8^+^ brain T_RM_ are significantly increased at 1 mo p.i (23, 24). We sought to determine if any of these cell populations remained elevated at 4 mo p.i to point towards an immunological explanation for the cognitive changes observed at that time. Flow cytometry was used to identify specific cell populations within the brain (**Supplemental Figure 4**), and numbers of cells/brain were quantified. Analysis of brain leukocytes 1 mo p.i. showed infection with *Lm* 10403s but not Δ*hly Lm* mutants triggered significant increases compared with uninfected mice of bone marrow-derived leukocytes in general, i.e. CD45^hi^ cells, as well as CD3^+^ T-lymphocytes and CD11b^+^ myeloid cells (**Figures 6A–C**). Quantification of the same leukocyte populations 4 mo p.i. revealed increased numbers of each population in uninfected mice compared with 1 mo p.i. This could be due to differences in technique, as the brains were not harvested at the same time, an effect of time, or both. Regardless, by 4 mo p.i., numbers of these cells in *Lm* 10403s infected mice were not different than found in uninfected mice harvested at the same time.

Next, we measured influxes of CD8^+^ and CD4^+^ T-lymphocytes and key sub-populations of these cells. Numbers of CD8^+^ and CD4^+^ T-lymphocytes were significantly increased 1 mo after infection with *Lm* 10403s (**Figures 6D, G**). In contrast, neither population was changed after infection with Δ*hly Lm* mutants. Notably, numbers of CD8^+^ T-lymphocytes remained significantly greater 4 mo after infection with *Lm* 10403s than those found in uninfected mice or in mice infected with Δ*hly Lm* mutants (**Figures 6D, G**). In contrast, numbers of CD4^+^ T-lymphocytes decreased in 10403s-infected mice to the degree that they were not different 4 mo after infection compared to uninfected or Δ*hly Lm* infected mice. Additionally, we quantified CD8^+^ and CD4^+^ T-lymphocyte sub-populations expressing the early activation marker CD69 (48) (**Figures 6E, H**) and CD8^+^ and CD4^+^ T_RM_ defined as CD62L^-^CD69^+^CX_3_CR1^-^CD103^+^ cells (24) (**Figures 6F, I**). CD69 was expressed by a greater percentage of CD8^+^ T-lymphocytes (70.2% ± 3.3%, mean ± SEM, n=12) than CD4^+^ T-lymphocytes (33.2% ± 1.1%, p < 0.0001). Numbers of CD8^+^ and CD4^+^ T-lymphocytes expressing CD69 and T_RM_ markers were significantly increased 1 mo after infection with *Lm* 10403s, but not Δ*hly Lm* mutants. Also, despite declining somewhat between 1 mo and 4mo, CD69^+^CD8^+^ T-lymphocytes and CD8^+^T_RM_ remained significantly increased at 4 mo. In contrast, numbers of CD69^+^CD4^+^ T-lymphocytes and CD4^+^ T_RM_ decreased notably from 1 to 4 mo p.i. and were no longer different from uninfected mice by 4 mo p.i.

**Figure 7:**
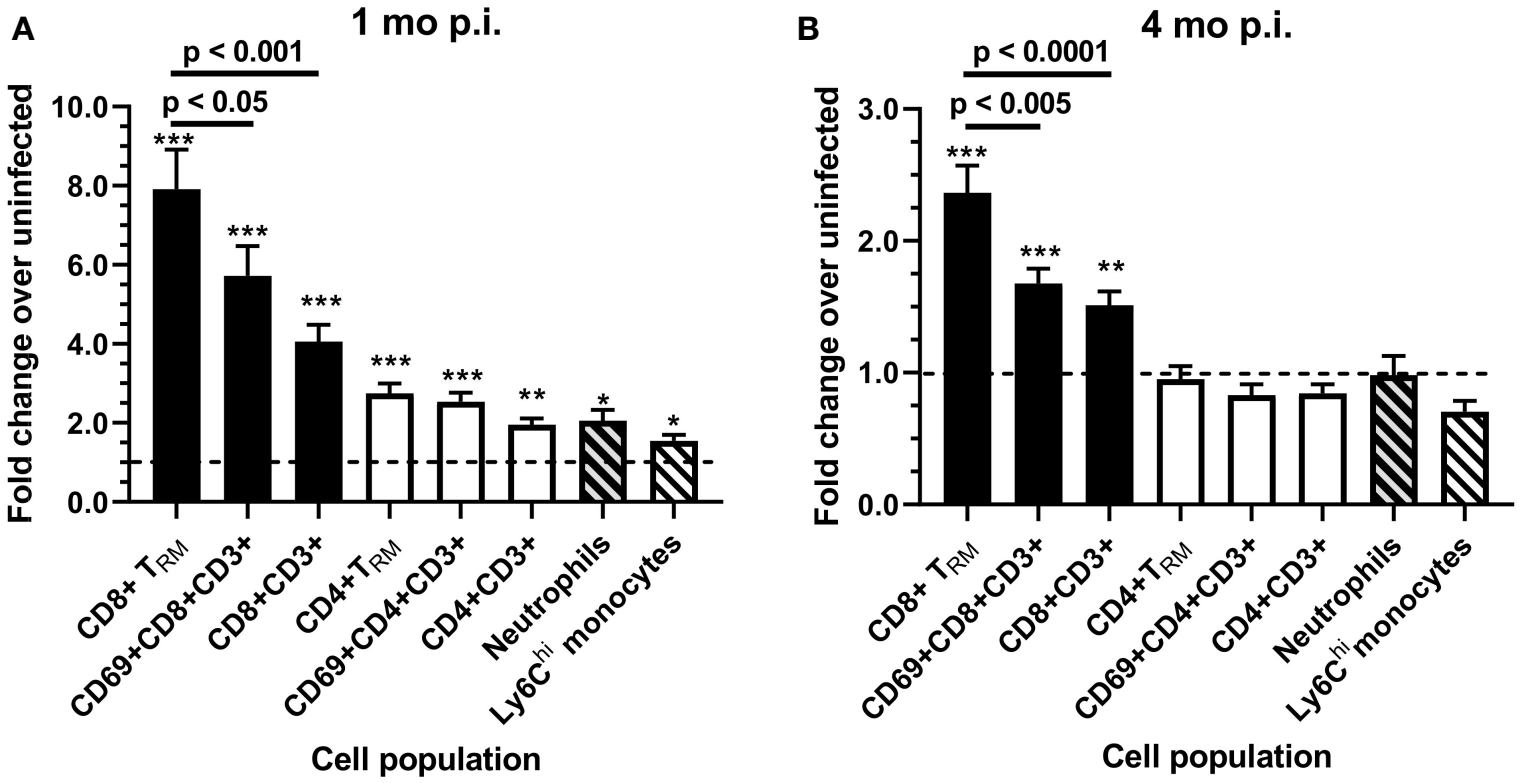
Fold changes of brain leukocytes 1 and 4 months after infection with *Lm* 10403s. Brain leukocytes collected from uninfected mice and from mice infected with *Lm* 10403s were quantified as described in **Figure 6**. Leukocytes from perfused brains were analyzed by flow cytometry and numbers of cells/brain calculated. Fold-changes for numbers of cells were calculated 1 mo **(A)** and 4 mo **(B)**, respectively, from infected mice (12, 12) over uninfected mice (6, 8) for each population and are represented as the mean + SEM fold change. Statistical differences between cell numbers from 10403s-infected and uninfected mice groups at 1 and 4 mo were calculated *via* 2-tailed Student’s *t*-test. *p < 0.05, **p < 0.01, ***p < 0.001. Significance among cell populations at the same time points were calculated *via* 1-way ANOVA with Tukey’s post-test and are shown.

Analyzing fold increases of each cell population in *Lm* 10403s infected mice over the respective cell numbers in uninfected mice harvested at the same time showed that CD8^+^ T_RM_ had the greatest fold increases 1 mo p.i. (7.91 ± 3.47) and 4 mo p.i. (2.74 ± 0.88) compared with other cell groups, including other CD8^+^ sub-sets (**Figure 7**). Infection-induced changes in cell numbers also resulted in a significant lowering of the brain CD4^+^/CD8^+^ T-lymphocyte ratio, decreasing from 1.04 ± 0.05 (mean ± SEM, n=6) in uninfected mice to 0.56 ± 0.06 (n=12) 1 mo after infection with *Lm* 10403s and from 0.79 ± 0.06 (n=8) in uninfected mice to 0.46 ± 0.04 (n=12) 4 mo after *Lm* 10403s infection. The CD4/CD8 ratio did not change significantly after Δ*hly Lm* infection (not shown).

Analysis of myeloid cells showed significant increases in numbers of granulocytes (fold increase 2.06 ± 0.95), and a trend for Ly6C^hi^ monocytes (p = 0.076) 1 mo after infection with *Lm* 10403s (**Supplemental Figures 5A, B**). Additionally, there was a small, but statistically significant increase in numbers of CD45^int^CD11^hi^ microglia in 10403s-infected mice (1.45 ± 0.32-fold) (**Supplemental Figure 5C**). This is similar to findings after sepsis induced in a cecal ligation and puncture model in which microgliosis has been noted (49). In contrast to the persistent elevation of CD8^+^ cells, numbers of granulocytes, Ly6C^hi^ monocytes, and microglia were the same in 10403s-infected mice as those found in uninfected mice by 4 mo p.i. (**Supplemental Figures 5B, C**). There were no changes in these cell populations 1 mo or 4 mo after infection with Δ*hly Lm*. Use of Ly6G^+^ to identify neutrophils specifically confirmed numbers of these cells were not increased in response to infection 4 mo p.i. (**Supplemental Figure 5D**).

Collectively these data show that 1 mo after infection with neuroinvasive *Lm*, numbers of several populations of bone marrow-derived leukocytes are significantly increased in the brain. These include CD4^+^ and CD8^+^ T-lymphocytes and sub-populations thereof with CD8^+^ cells showing the greatest magnitude of increase. In addition, numbers of neutrophils were increased. Of these however, only CD8^+^ cells, particularly CD8^+^T_RM_, remain elevated by 4 mo after infection. In contrast to *Lm* 10403s, infection with Δ*hly Lm* mutants exerted only minor effects on any cell population measured.

### Increasing numbers of brain CD8^+^ lineage T-lymphocytes correlates with worse performance in cognitive testing

Next, we performed a statistical evaluation of the relationship between specific leukocyte populations and results of studies obtained in the AHCoDA. AHCoDA measurements including independent learning index, reversal time to 80% success, cognitive flexibility, maximum learning, and reversal distance moved measured 1 month (**Table 1**) and 4 months (**Table 2**) after infection were correlated with numbers of different populations of brain leukocytes from the same mouse. Results from 1mo p.i. showed significant correlations only in one area. Specifically, higher numbers of CD45^hi^CD11b^+^ myeloid cells, as well as Ly6C^hi^ monocytes and neutrophils correlated significantly with a longer time to achieve the 80% success criterion. Including both cohorts 1 and 2 also showed significant correlation of CD45^hi^CD11b^+^ myeloid cells and neutrophils with increased time to achieve the 80% criterion but the correlation with Ly6C^hi^ monocytes was no longer significant (p=0.085). These results are consistent with the known role of neutrophils contributing to cognitive dysfunction after bacterial infection.

**Table 1:** Correlations and p values comparing AHCoDA measurements during reversal phase testing 1 mo after infection with brain cell populations.^1^.

Cell population	Independent Learning Index^2^		Reversal Time to 80%^3^		Cognitive Flexibility^2^		Maximum Learning^2^		Reversal Distance^2^	
	r	p	r	p	r	p	r	p	r	p
CD3^+^CD8^+^	-0.009	0.969	0.132	0.570	-0.009	0.969	0.058	0.801	0.001	0.996
CD8^+^CD69^+^	-0.131	0.571	0.103	0.658	0.012	0.960	0.114	0.622	0.032	0.889
CD8^+^T_RM_	-0.168	0.471	0.174	0.451	-0.051	0.827	0.091	0.695	0.049	0.832
CD3^+^CD4^+^	-0.213	0.357	0.040	0.865	-0.135	0.559	-0.043	0.854	0.016	0.947
CD4^+^CD69^+^	-0.317	0.162	0.125	0.589	-0.151	0.515	-0.095	0.683	-0.022	0.924
CD4^+^T_RM_	-0.182	0.430	0.093	0.689	0.179	0.437	0.005	0.982	-0.058	0.801
CD4/CD8	-0.098	0.670	-0.021	0.930	-0.130	0.575	-0.405	0.068	-0.127	0.583
Microglia	-0.230	0.316	-0.003	0.989	-0.325	0.151	-0.019	0.933	-0.240	0.294
CD45^hi^CD11b^+^	-0.262	0.251	**0.546**	**0.011**	-0.061	0.793	-0.074	0.750	-0.195	0.397
Ly6C^hi^ monocytes	-0.301	0.184	**0.480**	**0.028**	-0.030	0.898	-0.248	0.278	-0.087	0.708
Neutrophils	-0.234	0.308	**0.550**	**0.010**	-0.039	0.867	-0.184	0.424	-0.164	0.479

^1^Results of cognitive measurements from individual mice 4 months after infection with *Lm* 10403s (n=11), Δ*hly Lm* mutants (n=5), or antibiotics alone (n=5) were compared with results from quantification of specific cell populations by flow cytometry.

^2^Analysis by Spearman correlation with 2-tailed p value.

^3^Analysis by Pearson correlation with 2-tailed p value. Bold values are those that are statistically significant.

**Table 2:** Correlations and p values comparing AHCoDA measurements during reversal phase testing 4 mo after infection with brain cell populations.^1^.

Cell population	Independent Learning Index^2^		Reversal Time to 80%^3^		Cognitive Flexibility^2^		Maximum Learning^2^		Reversal Distance^2^	
	r	p	r	p	r	p	r	p	r	p
CD3^+^CD8^+^	**-0.559**	**0.027**	**0.530**	**0.035**	**-0.535**	**0.035**	**-0.597**	**0.017**	**-0.568**	**0.024**
CD8^+^CD69^+^	-0.360	0.180	**0.527**	**0.036**	-0.388	0.138	-0.376	0.151	**-0.571**	**0.023**
CD8^+^T_RM_	-0.359	0.173	**0.508**	**0.044**	-0.250	0.349	-0.359	0.173	**-0.647**	**0.008**
CD3^+^CD4^+^	0.085	0.755	-0.321	0.226	-0.094	0.730	0.012	0.969	0.277	0.299
CD4^+^CD69^+^	0.274	0.304	-0.324	0.105	0.082	0.763	0.165	0.541	0.218	0.417
CD4^+^T_RM_	0.377	0.151	-0.331	0.211	0.174	0.519	0.274	0.304	0.168	0.534
CD4/CD8	0.485	0.059	**-0.591**	**0.016**	0.253	0.343	0.403	0.123	**0.685**	**0.004**
Microglia	-0.097	0.721	-0.129	0.634	-0.147	0.586	-0.064	0.730	0.009	0.978
CD45^hi^CD11b^+^	-0.018	0.952	-0.201	0.456	-0.185	0.491	-0.077	0.780	0.309	0.244
Ly6C^hi^ monocytes	-0.160	0.564	-0.039	0.886	-0.227	0.400	-0.194	0.470	0.135	0.617
Neutrophils	-0.324	0.221	-0.008	0.976	-0.474	0.066	-0.318	0.230	0.009	0.978

^1^Results of cognitive measurements from individual mice 4 months after infection with Lm 10403s (n=5), Δ*hly* Lm mutants (n=4), or antibiotics alone (n=7) were compared with results from quantification of specific cell populations by flow cytometry.

^2^Analysis by Spearman correlation with 2-tailed p value.

^3^Analysis by Pearson correlation with 2-tailed p value. Bold values are those that are statistically significant.

Analyses derived from data 4mo after infection however, showed different correlations than at 1mo (**Table 2**). Increasing numbers of CD3^+^CD8^+^ T-lymphocytes correlated with lower independent index, longer time to meet the 80% success criterion, lower cognitive flexibility and maximum learning scores, and reduced distance moved during the reversal phase. Analyses of sub-sets of CD8^+^ T-lymphocytes showed significant correlations between increasing numbers of CD69^+^CD8^+^ T-lymphocytes and CD8^+^ T_RM_, as well as a CD4/CD8 skewed towards more CD8^+^ cells with longer time to meet the 80% success criterion, and reduced distance moved. In contrast, none of these parameters correlated significantly with CD4 lineage or myeloid cells. These findings support the hypothesis of a relationship between prolonged brain retention of CD8^+^ T-lymphocytes and post-infectious neuro-cognitive decline in this model.

## Discussion

*Lm* is a neuroinvasive bacterial pathogen commonly used to study innate and adaptive immune responses to CNS infection. Nonetheless, there is a notable lack of data regarding neuro-cognitive sequela after *Lm* infection. Data presented here show for the first time that mice recovered from *Lm* infection have deficits in several measurements of neuro-cognitive performance such as learning and extinction of learning compared with antibiotic-treated mice. Although subtle changes were observed 1 mo after infection, more significant and widespread declines were found 4 mo after infection. In accord with our hypothesis, we found cognitive decline significantly more pronounced after infection with neuroinvasive *Lm* strain 10403, which provoked persistent inflammatory cell recruitment to the brain, after infection compared to non-neuroinvasive Δ*hly Lm* mutants. Nonetheless, Δ*hly Lm-*infected animals also demonstrated measurable cognitive decline 4 mo after infection.

Neurocognitive studies in *Lm* infected mice were performed using an AHCoDA system with non-invasive monitoring. This minimizes both animal stress and experimenter contact with the animals, as well as variability introduced by these factors (50). AHCoDA is a highly sensitive technique that can detect subtle changes in behavior, and has been used to assess the effects of genetics (42), neuroactive compounds (51), gene mutations (39), and aging (38). This system incorporates tracking of spontaneous movement throughout light and dark cycles as well as the ability to assess cognitive flexibility by changing the food-reward algorithm. This procedure requires the mouse to extinguish its learning regarding how to obtain a food pellet from the initial discrimination phase and learn a new reward algorithm during the subsequent (reversal) phase of the study. Using this methodology, mice were tested for acquisition of spatial learning and reversal learning based on hundreds/thousands of entries (trials) that strengthens the robustness of the data. This contrasts with other testing methodology that characterize behavior with as few as 6-8 trials per mouse. Reversal learning is a more complex learning paradigm that involves the interaction of the hippocampal-based structures with multiple brain regions, including the orbitofrontal cortex, amygdala, and dorsal striatum (52). Our data show the impairments were present in initial and reversal learning, although were more pronounced during the reversal phase, suggesting that the hippocampal processing of spatial learning and neuronal communication between the different brain regions may be impaired. We also observed that *Lm* 10403s infected mice had significant declines in distance moved compared with uninfected mice. Interestingly, emerging data in human subjects shows that cognitive impairment is associated with reduced movement.

These and related data provide strong evidence that the mechanisms that contribute to cognitive impairment in humans are also active in the current animal model (47, 53). Results in our experiments are in striking accord with data from large observational studies of patients hospitalized for various infections and the risk of developing dementia. The key findings are that individuals hospitalized for CNS infections (bacterial or viral), as well as bacterial infections confined to the periphery, e.g. urinary tract, skin, and respiratory, had significantly greater risk of developing dementia over a prolonged observational period than did those with no hospitalizations for infection (7, 54, 55). Moreover, CNS infections inflicted the greatest risk of dementia with an adjusted hazard ratio (aHR) of 3.01 [95% CI 2.07 – 4.37], even though non-CNS infections also conferred an excess risk (aHR 1.47 [95% CI 1.36 – 1.59] (7). It is also notable that the risk of dementia after infection was significantly increased even when the infection-related hospitalization preceded the diagnosis of dementia by >2 yrs (54), >3 and <20 yrs (55), or > 10 yrs (7). Thus, dementia is a neuro-cognitive complication of infections severe enough to be hospitalized in addition to those already known to follow CNS infection (1, 2).

Experiments reported here compared neurocognitive activity and brain inflammation in mice after systemic infection with either neurovirulent *Lm* or non-invasive *Lm* Δ*hly* mutants to uninfected antibiotic-treated control mice. The *Lm Δhly* mutant is derived from the neuroinvasive 10403s strain and does not induce measurable influxes of Ly6C^hi^ monocytes, neutrophils, or lymphocytes after systemic injection (23, 34, 37). This strategy allows a more rigorous analysis of the role of brain inflammation compared to using uninfected mice only. Additionally, all mice received the same antibiotic treatment regimen regardless of infection status. This is important since antibiotics can have direct neuro-toxicity as well as indirect effects on behavior *via* alterations in the microbiome (56, 57). Another important control was measuring cells in each of these groups at the same time points, rather than simply comparing infected mice to a pre-injection/infection baseline. Numbers of homeostatic CD8^+^ brain T_RM_ have been reported to increase with aging (58, 59). Indeed, we identified small, but statistically significant increases between 1 mo and 4 mo in some cell populations, e.g. CD8^+^ T-lymphocytes & sub-populations, including CD8^+^ brain T_RM_, as well as microglia and granulocytes, but not in populations of CD4^+^ T-lymphocytes. It is possible that this reflects a real increase in bone marrow-derived cells in the brain, particularly CD8^+^ T-lymphocytes, but further confirmation is required to exclude technical issues and any effect of antibiotics.

A notable difference between this model of neuroinvasive *Lm* infection and other models of bacterial CNS infection is the density of bacteria within the CNS. For example, *Lm* infection initiated by systemic injection followed by antibiotics produces a bacterial load in the CNS <1,000 CFU *Lm*/brain, and neutrophilic infiltration is not a cardinal feature (23). Moreover, brains and spleens are typically sterile by 7d p.i (23, 24). In contrast, investigations of *S. pneumonia* meningitis frequently inject bacteria into the 3^rd^ ventricle causing rapid onset of meningitis, much larger bacteria loads e. g. ≥10^8^ CFU/brain or per ml CSF, and robust neutrophilic infiltration (60–62). Similarly, many models of viral CNS infection result in a much higher microbial load in the brain than the *Lm* model, and often target neurons (25, 63). In such models cognitive dysfunction is evident within 4 weeks of infection (62, 63). In our model, *Lm* infected mice showed minimal changes when analyzed 1 mo after infection supporting the idea that differences in pathogen load, cellular targeting by microbes, and characteristics of the host response can account for differential results in cognitive testing. In models of vascular cognitive impairment, cognitive changes are identified at later time points, e.g. 3-6 months post-insult (64, 65). This is also the case for the *Lm* infection model described here.

Analysis of brain leukocytes in *Lm* 10403s-infected mice showed significant brain influxes of CD4^+^ and CD8^+^ T-lymphocytes, including sub-populations expressing the CD69 activation marker and T_RM_ markers, as well as granulocytes, 1 mo after infection. However, by 4 mo p.i., only CD8^+^ cell populations remained significantly increased. In contrast, none of these leukocyte populations were altered after infection with Δ*hly Lm* mutants. CD8^+^ T-lymphocytes and CD8^+^ T_RM_ that infiltrate the brain in response to *Lm* infection produce IFN-γ and activate microglia (23, 31, 58). Moreover, other models have shown that these cells are clearly capable of instigating post-infectious cognitive dysfunction (26, 63, 66). Although functional studies of infiltrating brain cells were not performed here, we and others have shown that these cells are clearly capable of producing pro-inflammatory cytokines, e.g. IFN-γ and TNF (23, 31, 58). Indeed, we found increasing numbers of CD8^+^ T-lymphocytes significantly correlated with declines in several cognitive parameters including lower independent learning index, longer amount of time to reach the 80% success criterion, less cognitive flexibility and maximum learning, and decreased distance moved. In contrast, no such findings were present with CD4^+^ T-lymphocytes or sub-populations thereof, and the correlations were typically in the opposite direction as with CD8^+^ cells. Thus, CD8^+^ T-lymphocytes are likely to be key drivers of cognitive decline in mice recovered from neuroinvasive *Lm* infection.

Although CD8^+^ cells were significantly increased at both 1 mo and 4 mo p.i., but significant cognitive dysfunction was found only at 4 mo p.i. Thus, factors in addition to increased numbers of these cells must be involved to prevent or to effect cognitive dysfunction. A likely explanation is that time of exposure to these cells is a critical factor. This could be a feature of the *Lm* model due to the much lower degree of microbial infection in the brain (as detailed previously). Another possibility is that a population of short-lived regulatory cells are recruited into the brain by the infection and suppress early (i.e. 1 mo p.i.) immune-related cognitive dysfunction. For example, in some viral infection models, regulatory T-cells, Th2 cells, CD11c^+^ dendritic cells, and regulatory CD19^+^CD1d^hi^CD5^+^B- cells are recruited to the brain and limit CNS inflammation (67–70). Additionally, regulatory T-cells and Th2 cells recruited to the brain in experimental polymicrobial bacterial sepsis ameliorate some neuro-cognitive deficits measured 30d after induction of sepsis (71). It is possible that the numerically increased CD4^+^ population found 1mo p.i. in experiments reported here contained regulatory cells that prevented immune-related cognitive dysfunction at that time, but this positive effect waned as these cells returned to pre-infection levels by 4 mo p.i.

Further experiments are required to establish that CD8^+^T-lymphocytes drive cognitive dysfunction, the mediators responsible, and identify if regulatory cells that have a relevant role. These are directions for future experiments. Moreover, it is clear that not all of the cognitive dysfunction observed after *Lm* infection can be attributable to cellular infiltration in the brain. This was shown by impaired extinction of learning and distance moved 4 mo after infection with Δ*hly Lm* mutants despite the lack of measurable brain leukocytosis. The underlying mechanism likely results from systemic cytokines triggered in response to the infection with 10^7^ CFU bacteria, but this was not pursued here. Such a mechanism involving elevated systemic levels of TNF was proposed to cause accelerated cognitive decline in patients with Alzheimer’s disease (72). In support of this hypothesis, prior results showed injection of mice with Δ*hly Lm* mutants significantly increased concentrations of IFN-γ and TNF in serum 24hrs p.i., and increased expression of mRNA for CCL2 and CCL7 in the brain 48hrs p.i. and for TNF at 7d p.i (23, 37). Nonetheless, these bacteria do not induce influxes of monocytes or other cells into the brain when measured early, i.e. 48hrs or 7 days p.i (23, 37), or later at 28d p.i. (this paper).

It is clear that experiments with different strains of *Lm* could produce different results due to differential CNS invasion, virulence, or ability to or trigger inflammation (73–76). In this regard, we did not determine if brain inflammation in the absence of brain invasion also induces cognitive changes. Such an experiment could be performed with Δ*actA Lm* mutants that induce CNS inflammation in the absence of brain infection (23, 31, 37, 58). However, infection with Δ*actA Lm* also results in significantly lower CD8^+^ T-cell and CD8^+^ brain T_RM_ influxes than infection with wild type *Lm*, despite an approximate 20-fold greater inoculum (23, 58). Thus, achieving similar degrees of brain inflammation while controlling for other effects of the infection with mutated and virulent bacteria is challenging. Finally, because this study evaluated only male mice that were 8 weeks old when infected, results might not extend to female mice or to mice of other ages. This and the small numbers of animals may have limited our results.

## Conclusions

These findings demonstrate that cognitive decline follows systemic *Lm* infections that invade the brain and induce leukocyte influxes with retention of CD8^+^ T-lymphocytes in the brain. Moreover, less pronounced but still measurable cognitive decline can also be detected after infection with non-neuroinvasive *Lm* mutants that neither invade the brain nor induce prolonged leukocyte influxes. These novel results indicate two different mechanisms trigger post-infectious cognitive decline. One mechanism relies solely upon peripherally produced signals, likely proinflammatory cytokines, to trigger neuroinflammatory pathways in the brain that lead to cognitive decline. In contrast, the other may/may not combine these peripheral signals with the effects of CD8^+^ T-lymphocytes recruited into the brain during the acute infection and which are retained after bacteria have been eliminated. The ability to identify and measure these differences experimentally is a key step towards understanding the means by which different infections exert neuro-toxic actions and will give insight towards developing effective therapies for ameliorating cognitive decline after infection and other related conditions (77, 78).

## Data availability statement

The original contributions presented in the study are included in the article/**Supplementary Material**. Further inquiries can be directed to the corresponding author.

## Ethics statement

The animal study was reviewed and approved by Institutional Animal Care and Use Committee of the University of Oklahoma Health Sciences Center, Oklahoma City, OK, USA.

## Author contributions

BC designed and performed experiments, acquired flow cytometry data, analyzed and interpreted data, and drafted the manuscript and regulatory protocols. JF performed experiments. DO performed experiments, analyzed and interpreted data, performed troubleshooting. WS, SL and DD assisted with study design, data analysis and interpretation, and grant writing to perform the project. DD originated the project, supervised the studies and protocols for regulatory approval, and oversaw drafting and revision of the manuscript. All authors reviewed and critiqued the manuscript and approved the final manuscript. All authors contributed to the article and approved the submitted version.
