# Supplementary material for: Progressive cognitive impairment after recovery from neuroinvasive and non-neuroinvasive Listeria monocytogenes infection
Source: Front Immunol. 2023 Apr 18;14:1146690. doi: 10.3389/fimmu.2023.1146690 (PMC10151798; doi:10.3389/fimmu.2023.1146690)
Supplement: Supplementary file 1 [file DataSheet_1.docx]

Supplementary Material

**Progressive Cognitive Impairment after Recovery from *Listeria monocytogenes* Infection is Related to Retention of CD8^+^ T-lymphocytes in the Brain**

**Benjamin R. Cassidy^1#^, Sreemathi Logan^3^, Julie Farley^2^, Daniel Owen^2^, William E. Sonntag^2^, Douglas A. Drevets^1*^**

^1^ *Department of Internal Medicine, College of Medicine, University of Oklahoma Health Sciences Center, Oklahoma City, OK, United States,*

^2^*Department of Biochemistry and Molecular Biology, College of Medicine, University of Oklahoma Health Sciences Center, Oklahoma City, OK, United States,*

*Corresponding author:

Douglas A. Drevets, MD, DTM&H, FIDSA

Section of Infectious Diseases

800 Stanton L. Young, Suite 7300

Oklahoma City, OK 73104

Phone 405-271-6122

FAX 405-271-1570

Email: [douglas-drevets@ouhsc.edu](mailto:douglas-drevets@ouhsc.edu)

**Supplemental Fig. 1. Infection with *Lm* does not interrupt light/dark activity cycles.** Spontaneous movement over 89h was measured in uninfected mice (solid line), mice infected with *Lm* Δ*hly* mutants (dashed line) and *Lm* strain 10403s (dotted line) during Initial and Reversal phases at 1 mo p.i. (A) and 4 mo p.i. (B). Panels C-E show mean + SEM movement for each treatment group measured at 1 mo p.i. (solid line mean + SEM) compared with the same treatment group at 4 mo p.i. (dashed line).

**Supplemental Fig. 2. Reproducibility of cognitive testing results in different cohorts of *Lm* 10403s-infected mice.** A. C57BL/6J mice (age 2 mo) were injected i.p. with *Lm* 10403s, cohort 1 (□) received 3.2 x 10^6^ CFU whereas cohort 2 (▽) received 6.2 x 10^5^ CFU. All mice received antibiotics. Animals underwent cognitive testing in the Ethovision PhenoTyper with Cognition Wall. During hours 1-48 (Initial period), the mouse was rewarded by entering the left most entry of the cognition wall 5 times to receive a food pellet. The algorithm is reversed at hour 49 and from hours 49-89 (Reversal period), the mouse learned to enter the right most entry 5 times to receive a food reward. Movement was tracked using an IR camera located above the cage using Noldus Ethovision software. Results show the Independent learning index from individual mice in cohort 1 (□) and cohort 2 (▽), lines represent the mean +/- SEM. Panel B shows cohorts 1 and 2 combined (◇,⯁) and as compared with other mice receiving *Lm* Δhly (3.0 x 10^7^ CFU) or sterile PBS (●,○). As before, all mice received antibiotics beginning 2d after injection with the indicated bacteria or sterile PBS and underwent cognitive testing using the same testing paradigm. Results show the Independent learning index from individual mice, lines represent the group mean +/- SEM. 2-tailed t-test of panel A showed initial and reversal results of cohorts 1&2 were not different although the cohort 1 had greater variance in the initial period. Statistical analysis panels B-D by ordinary one-way ANOVA with multiple comparisons post-test via two-stage linear step-up procedure of Benjamini, Krieger and Yekutieli. Discoveries (q < 0.05) in panel B comparing groups to Uninfected mice in the Initial period are marked by an (*). For panels C and D, discoveries (q < 0.05) comparing dark cycles 2-4 to dark cycle 1 within a group are indicated by an (*), discoveries across other groups are shown.

**Supplemental Fig. 3. Measurements of cognitive flexibility and maximum learning after infection with virulent and avirulent *Lm***. Cognitive Flexibility (A, C) and Maximum Learning (B, D) were measured in uninfected mice (open columns) 1 mo (n = 12) and 4 mo (n=7), and in mice infected with *Lm* Δ*hly* mutants (hatched columns) at 1 mo (n=9) and 4 mo (n=7) p.i., and *Lm* 10403s-infected mice (black columns) at 4 mo (n=5). Data for *Lm*10403s-infected mice at 1 mo in panels A and B include only cohort 1 (n=11) whereas panels C and D include cohorts 1 and 2 (n=21), Columns show mean + SEM for all groups. P values between the same treatment groups at 1 and 4 mo p.i. were calculated by 2-tailed t-test and are given.

**Supplemental Fig. 4. Gating of brain cells.** Brain leukocytes were incubated with fluorochrome-labeled mAb or isotype control mAb, then analyzed on a Stratedigm S1200Ex flowcytometer. Positive staining was established by comparing non-specific staining of similar cell populations derived from a limited panel of mAb, e.g. CD45 and CD11b only to identify CD45^hi^CD11b^-/lo^ cells, with all other mAb being fluorochrome-labeled isotype controls. Initial gates consisted of cell gates to avoid debris, and cell clumps of cells (not shown). Specific populations of cells, and their gates are shown. Gating of total bone marrow-derived cells (CD45^hi^) is shown in (A). Bone marrow-derived myeloid cells were first identified as CD45^hi^CD11b^hi^ (B) cells. Ly6C^hi^ monocytes were identified as SSC^lo^Ly6C^hi^ (C) or Ly6G^-^Ly6C^hi^ (D) cells. Granulocytes and neutrophils were identified as SSC^ji^Ly6C^int^ (C) or Ly6G^+^Ly6C^int^ (D) cells, respectively. Microglia were identified as CD45^int^CD11b^hi^ (B). Tissue resident memory lymphocytes were first identified CD45^hi^CD11b^-/lo^ (B) then as CD3^+^ (E) and either CD8^+^ or CD4^+^ (F) in addition to being CD69^+^CD62^-^ (G, I) and CX3CR1^-/lo^CD103^+^ (H, J). Histograms show gates defining CD103^+^ (K) and CX_3_CR1^-/lo^ (L) lymphocytes. CD45^hi^ brain leukocytes incubated with isotype control mAb are shown by shaded histograms, whereas CD3^+^CD8^+^CD69^+^CD62L^-^  lymphocytes labeled with CD103 (K) or CX_3_CR1 (L) are shown by solid lines. Additionally, CX_3_CR1 labeling of CD45^hi^ brain leukocytes (dashed line) is presented in (L).

**Supplemental Fig. 5 Analysis of myeloid cell populations after *Lm* infection.** A-C Brain leukocytes collected from uninfected mice (●, ○) and from mice infected with *Lm* Δ*hly* mutants (▲, Δ) and *Lm* 10403s (■, □) as described in Fig. 1, along with additional mice from the same cohorts for which cognitive data was not collected. Leukocytes were collected from perfused brains and analyzed by flow cytometry. Symbols indicate individual mice and lines represent group median. Numbers of samples used at 1 mo (A) and 4 mo (B), respectively, from uninfected mice (6, 8) *Lm* 10403s infected mice (12, 12) and *Lm* Δ*hly* mutants (5, 6). Neutrophils were defined as CD45^hi^CD11b^hi^Ly6C^int^Ly6G^+^ cells in mice 4 mo post-injection from uninfected mice (○) and from mice infected with *Lm* Δ*hly* mutants (Δ) and *Lm* 10403s (□). Symbols indicate individual mice and lines represent group mean +/- 95% CI. Statistical significance by ANOVA among groups at 1 mo or 4 mo p.i. is shown. Significance via 2-tailed Student’s *t*-test between the same challenge groups at 1 and 4 mo are shown as * p < 0.05, ** p < 0.01, *** p < 0.001.
